# Editorial: Local Aspects of Sleep and Wakefulness

**DOI:** 10.3389/fnins.2020.00058

**Published:** 2020-02-07

**Authors:** Giulio Bernardi, Francesca Siclari, Michele Bellesi

**Affiliations:** ^1^IMT School for Advanced Studies Lucca, Lucca, Italy; ^2^Lausanne University Hospital (CHUV), Lausanne, Switzerland; ^3^School of Physiology, Pharmacology & Neuroscience, University of Bristol, Bristol, United Kingdom

**Keywords:** sleep, hd-EEG, MEG, fMRI-EEG, K-complex, slow wave, spindle, connectivity

In the last two decades, the traditional view of sleep and wakefulness as global, whole-brain states has been challenged by a growing body of evidence indicating that both sleep and wake are regulated locally and that sleep- and wake-like activity can often co-occur across distinct brain areas. This change in perspective has led to a better understanding of sleep physiology and pathophysiology. The present Research Topic highlights current research and views on the local regulation of sleep and wakefulness in multiple species, models and systems. The articles, which include both original research and reviews, describe results obtained using a variety of experimental techniques and analytical methods.

Several articles explore the local aspects of sleep hallmarks, including slow waves, K-complexes (KCs) and spindles, and evaluate how they could relate to typical features and known functions of sleep, including sensory disconnection and memory consolidation. Using magnetoencephalographic (MEG) recordings in N2-sleep, Ioannides et al. demonstrate that KCs, commonly considered as “global” events, are characterized by important inter-regional differences. The authors show that spontaneous KCs are associated with changes in brain activity that mostly involve medial frontal and cingulate areas, whereas more lateral and posterior regions are usually spared. In addition, their results indicate that KCs are preceded by specific activity changes that are circumscribed to the dorsal caudal anterior cingulate cortex. Laurino et al. complement these observations through the study of sensory evoked KCs. Using source modeling of high-density electroencephalographic (hd-EEG) recordings, they show that the first component of KCs is a positive signal deflection with a specific cortical distribution that depends on the nature of the administered sensory stimulus. This positive wave, which is associated with an increase in high-frequency activity, appears to travel toward frontal brain regions where the main negative component of the KC is eventually ignited.

Other two studies focused on the local correlates of sleep spindles. Specifically, Alfonsi et al. used source modeling of scalp EEG data to identify the cortical sources of sleep spindles and their variations across a night of sleep. They confirm that faster spindles predominate over parietal cortical areas, while slower spindles involve more frontal regions. Moreover, the authors show that activity of frontal slow spindles tends to decrease from the beginning to the end of the night, while, within each cycle, activity of both frontal and parietal spindles follows a U-shaped curve. Fang et al. used simultaneous fMRI-EEG to study cortical and subcortical areas involved in sleep spindles and to evaluate their potential relationship with cognitive skills. They report activations in the thalamus, bilateral striatum, middle cingulate cortex, and cerebellum. Further, spindle activation in a subset of these structures correlated with reasoning abilities, but not short-term memory or verbal abilities.

In their opinion piece, Geva-Sagiv and Nir outline available evidence linking local changes in slow waves and spindles to experience-dependent brain plasticity and learning. They show how cortical and thalamic sleep oscillations are coupled with hippocampal activity and may thus support memory consolidation. The authors also describe how recent discoveries of novel tools allowing to artificially modulate sleep oscillations could potentially be used to modulate sleep-dependent memory processing. Finally, they underscore the need to achieve a better understanding of how the local regulation and co-regulation of slow waves and spindles relates to changes in hippocampal activity.

The local regulation of sleep may have important implications not only for experience-dependent brain plasticity, but also for the maintenance of an efficient sensory disconnection during sleep. Tamaki and Sasaki used source localization of hd-EEG recordings to confirm and extend their recent observation of an inter-hemispheric asymmetry in NREM slow wave activity during the first night of sleep in an unfamiliar environment. Interestingly, they show that while a similar asymmetry in brain activity is not present during REM sleep, in phasic REM periods the amplitude of evoked brain responses to deviant tones is greater during the first relative to the second night. These findings suggest the presence of distinct brain mechanisms to monitor an unfamiliar environment during NREM and REM sleep.

Two review articles highlight the importance of employing distinct animal models to achieve a deeper understanding of the local regulation of sleep and wakefulness. Rattenborg et al. describe similarity and differences in local sleep regulation across different species of birds and mammals. While evidence clearly indicates that wakefulness, NREM and REM sleep often manifest as non-discrete states in many different species, inter-species differences and peculiarities may hold the key to understand the functions and mechanisms of sleep-related brain activity. Moreover, Vantomme et al. outline evidence from animal studies supporting a possible role of the thalamic reticular nucleus (TRN) in the local regulation of sleep oscillations. In fact, while recent research especially focused on the cortical mechanisms of local sleep regulation, the involvement of subcortical structures is still poorly investigated and understood. Here, Vantomme and colleagues show that subcortical circuits may contribute to the tuning of regional sleep patterns and suggest that alterations of such circuits could potentially explain aberrant local changes in cortical brain activity observed in distinct pathological conditions.

The falling asleep and the awakening processes represent transitional states associated with changes in activity that do not occur simultaneously across all brain regions. The study of these states may thus provide essential information regarding the local cortical and subcortical regulation of sleep. For the first time, two studies explore here how brain activity and connectivity change at sleep onset following a period of acute sleep deprivation. Gorgoni et al. show that recovery sleep is characterized by a stronger and more diffuse frontal increase in low-frequency activity relative to sleep onset in baseline conditions. In contrast, changes in sigma and beta activity, respectively characterized by an increase over centro-parietal areas and a widespread decrease, are scarcely affected by sleep loss. In addition, Fernandez Guerrero and Achermann show that sleep onset is characterized by an anterior-to-posterior decoupling within the so-called default mode network (DMN), while increased connectivity from posterior cingulate cortex to other nodes of the DMN is observed for most frequencies. Increased sleep drive caused by sleep deprivation leads to similar changes, but with decreased connectivity among several pairs of areas, especially in the high-frequency range.

The fact that sleep is locally regulated implies that islands of sleep-like activity could also appear during behavioral and electrophysiological wakefulness. Two review papers describe the physiological meaning and possible behavioral and cognitive impact of these “local sleep” episodes during periods of waking. D'Ambrosio et al. summarize the neurophysiological bases of local sleep and compare it to other forms of intrusions of sleep into wakefulness, such as microsleep episodes. Importantly, local sleep episodes increase in number and extension with time spent awake and have a proven negative impact on behavioral performance. Thus, the authors suggest that local sleep may represent a signature of brain fatigue, potentially accounting for many of the cognitive and behavioral manifestations of sleepiness. Andrillon et al. further suggest that local sleep intrusions could affect subjective experience associated with attentional lapses, leading to either mind wandering or mind blanking. Their proposed framework may have important implications for our understanding of inter-individual differences in attentional lapses in physiological and pathological conditions, such as attention deficit hyperactivity disorder (ADHD). In fact, alterations in the local regulation of sleep and wakefulness could contribute to explain still poorly understood symptoms of many pathological conditions. In line with this, Christensen et al. demonstrate that insomnia patients are characterized by a stronger expression of sleep patterns characteristic of light sleep or wakefulness, even during the deepest stages of sleep, indicating a deep sleep fragility.

Finally, in their work Héricé and Sakata show that even a simple network model of the sleep–wake cycle can be associated with complex behaviors. Their results indicate the importance of carefully taking into account the complexity of sleep-wake regulation circuits when designing *in vivo* experiments and interpreting their results, but also show the fundamental complementary role of computational models in studying the physiological mechanisms regulating sleep-related brain activity.

Overall, this Research Topic underscores the importance of combining multiple experimental models and approaches to broaden our understanding of sleep regulation. The editors hope that the papers published herein will stimulate further investigations aimed at elucidating the complex mechanisms underpinning the local regulation of sleep and wakefulness, and their implications for behavior and cognition in both physiological and pathological conditions.

## Author Contributions

GB wrote the first draft. All authors revised and edited the manuscript.

### Conflict of Interest

The authors declare that the research was conducted in the absence of any commercial or financial relationships that could be construed as a potential conflict of interest.

